# First-passage-time statistics of growing microbial populations carry an imprint of initial conditions

**DOI:** 10.1038/s41598-023-48726-w

**Published:** 2023-12-04

**Authors:** Eric W. Jones, Joshua Derrick, Roger M. Nisbet, William B. Ludington, David A. Sivak

**Affiliations:** 1 Department of Physics, Simon Fraser University, Burnaby, BC V5A 1S6 Canada; 2 Department of Biological Sciences and Engineering, Carnegie Institution for Science, Baltimore, MD 21218 USA; 3 Department of Ecology, Evolution, and Marine Biology, University of California, Santa Barbara, Santa Barbara, CA 93106 USA; 4 Department of Biology, Johns Hopkins University, Baltimore, MD 21218 USA

**Keywords:** Biological physics, Population dynamics, Statistical physics

## Abstract

In exponential population growth, variability in the timing of individual division events and environmental factors (including stochastic inoculation) compound to produce variable growth trajectories. In several stochastic models of exponential growth we show power-law relationships that relate variability in the time required to reach a threshold population size to growth rate and inoculum size. Population-growth experiments in *E. coli* and *S. aureus* with inoculum sizes ranging between 1 and 100 are consistent with these relationships. We quantify how noise accumulates over time, finding that it encodes—and can be used to deduce—information about the early growth rate of a population.

## Introduction

Bacteria divide, viruses replicate, and yeast cells bud, leading (if unimpeded) to exponential growth. Since division events are generally not evenly separated in time, even identically prepared systems will give rise to variable growth trajectories. Unconstrained environmental factors like stochastic inoculation further amplify this variability. Traditionally, the study of noisy population growth has maintained a focus on population abundance^1,2^; for example, the 1943 Luria-Delbrück experiment used variation in the abundance of phage-resistant bacteria at a given time to deduce that bacteria mutate independent of selective pressures^3^.

In this paper we offer an alternative approach by characterizing noisy population growth in terms of a population’s temporal variation, specifically the temporal standard deviation (TSD), the standard deviation of the distribution of times at which a growing population first hits a threshold number. We apply stochastic models of exponential growth to relate the TSD at large thresholds to the inoculum size and growth rate, deriving power-law relationships that match direct experimental tests in *Escherichia coli* and *Staphylococcus aureus*.

The processes of bacterial growth and division have been extensively modeled^4–10^ and empirically characterized^11,12^ over the past century. Especially over the last 15 years, experiments that enable the high-throughput, long-term observation of bacteria^13,14^ have advanced the fine-grained modeling of bacterial division^15–17^. In this paper, we propose that temporal variation is a natural lens for examining and quantifying the noisy growth of replicate bacterial populations.

The initial conditions of a population affect its subsequent growth. Conversely, statistics of noisy abundance trajectories report on a population’s early growth conditions, though the precise interpretation of these statistics depends on a specified stochastic growth model. We first analyze two analytically tractable models of exponential growth: (i) the simple birth process, perhaps the most basic stochastic model of exponential growth, which assumes that each individual divides according to a Poisson process; and (ii) a model in which inoculum sizes are drawn from a Poisson distribution and growth dynamics are deterministic. Identical power-law relationships between TSD, inoculum size, and growth rate are derived for these two models. Then, we numerically examine age-structured population-growth models that account for an organism’s age. Last, we present bacterial growth experiments that complement and empirically ground these power-law relationships, demonstrating that statistics reporting on the temporal variation provide practical biological insights.

**Figure 1:**
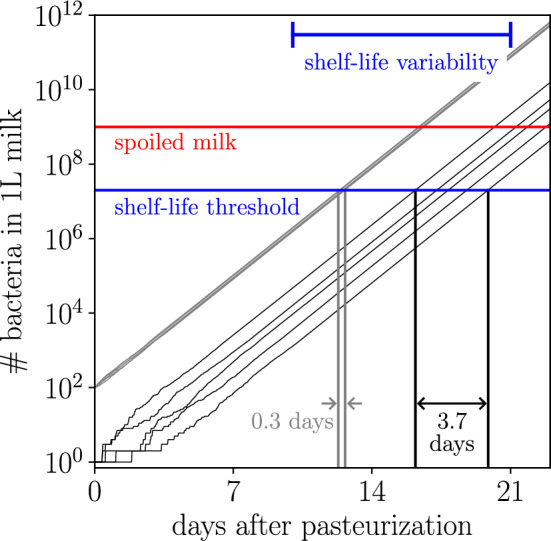
Intrinsic variability contributes to the reported 11-day variation in the shelf life of milk. Abundance trajectories from a simple birth process modeling the growth of *L. monocytogenes*, a common milk contaminant that divides roughly every 17 h, inoculated with a single individual (black) or 100 individuals (gray). The measured 10–21 day shelf life of milk is reported in Ref^18^.

As a tangible example, consider milk spoilage^19–21^. Milk spoilage occurs when the exponential growth of a contaminant bacteria reaches some threshold population density. In a refrigerator at 5 °C, the common bacterial contaminant *Listeria monocytogenes* divides every $\sim$17 h^22^. It is straightforward to measure the distribution of times at which a number of identically prepared containers of milk spoil: if properly refrigerated, pasteurized milk has a shelf life (time to reach a bacterial concentration of 20,000 CFU/mL^23^) ranging from 10 to 21 days post processing^18^. Figure 1 shows simulated abundance trajectories for the simple birth process modeling the growth of *L. monocytogenes*, which indicate that a liter of milk inoculated by a single bacterium has a shelf life of roughly 17 days with a 3.7-day range, while a liter of milk inoculated by 100 bacteria has a shelf life of 12 days with a 0.3-day range. Nearly 4 days of the variation in the timing of milk spoilage can be accounted for by the simple birth process. The remaining variation must be generated by other environmental factors. Food-processing engineers that decompose noise into its constitutive processes might learn whether variability is inevitable or whether it can be mitigated.

## Results

### Models of exponential growth

#### Simple birth process

First, consider a simple birth process in which each individual divides according to a Poisson process with rate $\mu$. This model was first solved in 1939, then subsequently used as a model of bacterial growth^4,24,25^. This analytically tractable model permits direct calculation of statistics that report on the population’s temporal variation, namely the temporal variance $\sigma ^2_t$ and the temporal standard deviation $\sigma _t$ (TSD).

For a population of *n* individuals, the probability $B_n$ per unit time that an individual will divide (conventionally the “birth rate” in Markov-process literature^26^) is

1 $$\begin{aligned} B_n = \mu n. \end{aligned}$$

The probability $P_t(n \, | \, n_0)$ that the population consists of *n* individuals at time *t*, given an inoculum of $n_0$ individuals, is governed by the master equation

2 $$\begin{aligned} \frac{\text {d}}{\text {d} t} P_t(n \, |\, n_0) = \mu (n-1) P_{t}(n-1 \, | \, n_0) - \mu n P_t(n \, | \, n_0). \end{aligned}$$

In $P_t(n \, | \, n_0)$, *n* is the random variable with normalization $\sum _{n=0}^\infty P_t(n \, | \, n_0) = 1$. Using generating functions^4,24^, the solution is

3 $$\begin{aligned} P_{t}(n \, | \, n_0)&= {n-1 \atopwithdelims ()n_0-1} e^{-\mu n_0 t} (1 - e^{-\mu t})^{n-n_0}, \end{aligned}$$

for binomial coefficient ${i \atopwithdelims ()j} \equiv i!/j! (i-j)!$. The first two cumulants are the average abundance

4 $$\begin{aligned} \langle n \rangle = n_0 e^{\mu t}, \end{aligned}$$

which grows exponentially, and the variance

5 $$\begin{aligned} \langle (n - \langle n \rangle )^2\rangle = n_0 e^{\mu t}(e^{\mu t} - 1). \end{aligned}$$

The first-passage-time distribution $P_{\Omega }^{\text {FP}}(t \, | \, n_0)$ is the distribution of times at which a population with inoculum size $n_0$ first reaches $\Omega$ individuals^27^. Since the simple birth process yields monotonic abundance trajectories, the reaction probability $R_{\Omega }(t \, | \, n_0)$ that at time *t* the population size is greater than or equal to population threshold $\Omega$ is related to the first-passage-time probability $P_{\Omega }^{\text {FP}}(t \, | \, n_0)$:

6 $$\begin{aligned} R_{\Omega }(t \, | \, n_0)&= 1 - \sum _{i=n_0}^{\Omega -1} P_t(i \, | \, n_0) =\int _0^t P_{\Omega }^{\text {FP}}(\tau \, | \, n_0) \, \text {d}\tau . \end{aligned}$$

Therefore,

7 $$\begin{aligned} P_{\Omega }^{\text {FP}}(t \, | \, n_0) = - \sum _{i=n_0}^{\Omega -1} \frac{\text {d} P_t(i \, | \, n_0)}{\text {d}t}, \end{aligned}$$

yielding (Supplementary Information, Section A)

8 $$\begin{aligned} P_{\Omega }^{\text {FP}}(t \, | \, n_0)&= \mu (\Omega -n_0) {\Omega -1 \atopwithdelims ()n_0 - 1} (e^{-\mu t})^{n_0}(1 - e^{-\mu t})^{\Omega -n_0-1} . \end{aligned}$$

The mean first-passage time $\langle t \rangle _{\Omega \, | \, n_0}$ to reach threshold $\Omega$ starting from $n_0$ individuals is (SI, Section B)

9a $$\begin{aligned} \langle t \rangle _{\Omega \, | \, n_0}&= \frac{1}{\mu }\left( \frac{1}{n_0} + \frac{1}{n_0 + 1} + \cdots + \frac{1}{\Omega -1} \right) , \end{aligned}$$

9b $$\begin{aligned}&\approx \frac{\ln \Omega }{\mu } - \frac{\ln n_0}{\mu } \quad \text {for large } \Omega \gg n_0. \end{aligned}$$

The *temporal variance* $\sigma ^2_t \equiv \left\langle (t - \langle t\rangle )^2 \right\rangle _{\Omega \, | \, n_0}$ is (SI, Section B)

10 $$\begin{aligned} \sigma _t^2 = \frac{1}{\mu ^2}\left[ \frac{1}{n_0^2} + \frac{1}{(n_0+1)^2} + \cdots + \frac{1}{(\Omega -1)^2}\right] , \end{aligned}$$

and therefore the *temporal standard deviation* is

11a $$\begin{aligned} \sigma _t&= \frac{1}{\mu }\left[ \frac{1}{n_0^2} + \frac{1}{(n_0+1)^2} + \cdots + \frac{1}{(\Omega -1)^2}\right] ^{1/2} \end{aligned}$$

11b $$\begin{aligned}&\approx \frac{1}{\mu n_0^{1/2}} \quad \text {for large } \Omega \gg n_0. \end{aligned}$$

**Figure 2:**
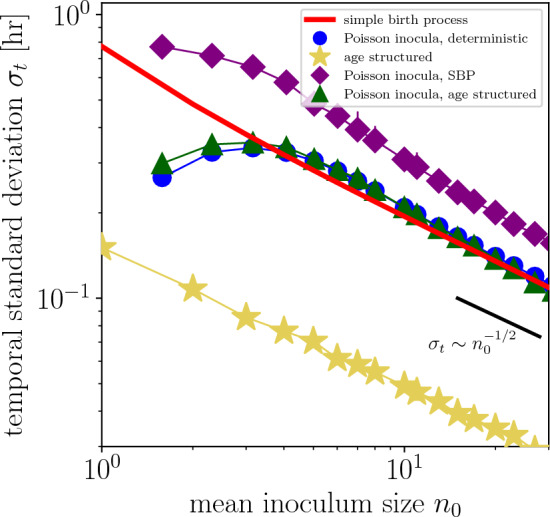
Temporal standard deviation (TSD) scales inversely with the square root of inoculum size for five models of stochastic exponential growth. For each model, inocula are either exact or Poisson-distributed, and growth either obeys the simple birth process (SBP), deterministic exponential growth, or age-structured growth. The growth rate $\mu$ for the simple birth process and deterministic growth is 1.66/hr, corresponding to a 25-min division time. The division-time distribution for the age-structured population-growth model has a 25-min mean division time and a 22% coefficient of variation (Fig. S1). At least $n=2,000$ replicates were simulated for each model and inoculum size. Error bars, which are typically smaller than the corresponding symbol, show 95% confidence intervals (Methods). For Poisson-distributed inocula, the x-axis reports the zero-truncated mean inoculum size. Lines are a guide to the eye.

This exact relationship between TSD, growth rate, and inoculum size for the simple birth process is plotted in Fig. 2 (red curve).

The mean first-passage time (9a) and the temporal variance (10) can alternatively be solved by leveraging the Markovianity of the simple birth process: a population of size *n* experiences an exponentially distributed waiting time with mean $1/\mu n$ before an individual in the population divides, and the variance of this waiting-time distribution is $1/(\mu n)^2$. Waiting times are independent, so moments of the first-passage-time distribution are simply the sum of the moments of the waiting-time distributions. However, this approach does not immediately provide the first-passage-time distribution Eq. (8).

#### Poisson-distributed inocula undergoing deterministic exponential growth

Departing from the assumption that populations are initialized with exactly $n_0$ individuals, we next consider populations with Poisson-distributed inocula that grow deterministically. This scenario is relevant because bacterial inoculation in our experiments—performed by pipetting a fixed volume of a dilute solution of bacteria—resulted in Poisson-distributed inocula (Fig. S2). Populations with Poisson-distributed inocula are more variable than populations that are exactly inoculated, as variability in the inoculum size propagates through the growth dynamics.

As before, replicate populations give rise to a distribution of abundance trajectories. We exclusively consider trajectories with nonzero inoculum sizes such that the probability $P_{n_0}(k)$ of starting with *k* individuals is

12 $$\begin{aligned} {P}_{n_0}(k) = \frac{e^{-n_0} n_0^k}{k! (1 - e^{-n_0})}, \end{aligned}$$

corresponding to mean inoculum size $n_0/(1-e^{-n_0})$ for Poisson shape parameter $n_0$.

We consider deterministic population growth

13 $$\begin{aligned} n(t) = k e^{\mu t}, \end{aligned}$$

a simplifying assumption that implies the abundance *n*(*t*) takes on non-integer values. The random variable

14 $$\begin{aligned} T(M) \equiv \frac{1}{\mu } \ln (\Omega /M) \end{aligned}$$

is the first-passage time at a threshold $\Omega$ given that the inoculum size is a random variable *M*. The temporal standard deviation can be computed exactly, albeit opaquely:

15 $$\begin{aligned} \sqrt{\langle T(M)^2 \rangle - \langle T(M) \rangle ^2}&= \left\{ \frac{e^{-n_0}}{\mu ^2 (1 - e^{-n_0})} \left[ \sum _{k=1}^\infty \frac{ (\log {k})^2 \, n_0^k}{k!} - \left( \sum _{k=1}^\infty \frac{n_0^k \, \log {k}}{k!} \right) ^2 \frac{e^{-n_0}}{1-e^{-n_0}} \right] \right\} ^{1/2}. \end{aligned}$$

This temporal standard deviation is plotted as a function of mean inoculum size in Fig. 2 (blue circles).

To obtain the TSD at large $n_0$, first note that for large $n_0$ the Poisson distribution Eq. (12) is well-approximated by a normal distribution with mean $n_0$ and variance $n_0$, and the quantity $1 - e^{-n_0}$ is well-approximated by 1. Then, the “delta method”^28,29^ gives access to the mean and variance of the random variable *T*(*M*) in terms of cumulants of *M*:

16a $$\begin{aligned} \langle T(M) \rangle&= T( \langle M \rangle ) + \frac{T''(\langle M \rangle )}{2} \left( \langle M^2 \rangle - \langle M \rangle ^2 \right) + \text {higher-order terms} \end{aligned}$$

16b $$\begin{aligned}&= \frac{\log (\Omega /n_0)}{\mu } + \frac{1}{2 \mu n_0} + O\left( \frac{1}{n_0^2} \right) , \end{aligned}$$

and

17a $$\begin{aligned} \langle T(M)^2 \rangle - \langle T(M) \rangle ^2&= [T'(\langle M \rangle )]^2 \left( \langle M^2 \rangle - \langle M \rangle ^2 \right) + \text {higher-order terms} \end{aligned}$$

17b $$\begin{aligned}&= \frac{1}{\mu ^2 n_0} + O\left( \frac{1}{n_0^2} \right) , \end{aligned}$$

where the higher-order terms depend on third and higher cumulants of *M* that vanish when *M* is normally distributed.

Therefore, for large $n_0$, TSD and inoculum size are related by:

18 $$\begin{aligned} \sqrt{\langle T(M)^2 \rangle - \langle T(M) \rangle ^2} \approx \frac{1}{\mu n_0^{1/2}}. \end{aligned}$$

This is the same relationship between TSD, inoculum size, and growth rate as for the simple birth process with exact inoculation, Eq. (11b).

#### Age-structured population growth

Organismal division is carefully choreographed, and we next turn to models that resolve some of the structure of individual division events. We performed agent-based simulations of age-structured population growth in which division-time distributions fully describe the timing of division events (Methods). To be precise, this model is a type of Bellman-Harris stochastic branching process^30^. We used an approximately normal division-time distribution with 25-minute mean and 22% coefficient of variation^4^. Inoculated individuals were assumed to be at a random time along their division cycle.

From these simulated abundance trajectories, TSDs were evaluated at a threshold of 500 individuals and are plotted as gold stars in Fig. 2. While the more complicated structure of this population-growth model prevents analytic examination, the scaling of TSD with inoculum size visually follows the -1/2 power law predicted by the simple birth process and by Poisson-distributed inocula with exponential growth.

#### Comparing models of population growth

Last, we simulated models for every combination of inoculation (exact or Poisson-distributed) and population growth (simple birth process, deterministic, or age-structured) (Methods). Figure 2 shows numerically calculated TSDs for Poisson-distributed inocula obeying the simple birth process (purple diamonds), and for Poisson-distributed inocula undergoing age-structured growth (green triangles).

The models showcased in Fig. 2 ostensibly describe the same organism, but differ in their biological assumptions about inoculation and growth. The relationships between TSD and inoculum size quantify the effects of these assumptions on observed temporal variation. In particular, we found that the relationship between TSD and inoculum size for a biologically faithful model that captured stochasticity in inoculation and growth (green triangles) was similar to the relationship for the simple birth process (red line).

The mean trajectories of the different stochastic growth models—unlike the temporal variation—are nearly indistinguishable for a given inoculum size, highlighting an advantage of noise-based analyses. For example, TSDs for age-structured growth are $\sim$5 times smaller than for the simple birth process, a consequence of the fact that tighter division-time distributions give rise to less variable growth trajectories^4^. Especially for organisms with constrained division-time distributions, the noise from Poisson inoculation dominates the noise due to growth, which explains why the blue circles and green triangles are so similar in Fig. 2. For exactly inoculated populations, broadening the age-structured division-time distribution from 22% coefficient of variation to 100% interpolates between the gold stars and red line; similarly, for Poisson-distributed inocula, it interpolates between the green triangles and purple diamonds.

Temporal variances approximately add: the temporal variance of populations with Poisson-distributed inocula that follow the simple birth process is roughly the sum of the temporal variance of exactly inoculated populations growing according to the simple birth process and the temporal variance of populations with Poisson-distributed inocula and deterministic growth.

We have used mathematical models of varying resolution to describe population growth, trading off biological realism for analytic tractability. For example, the simple birth process assumes that a bacterium’s age is irrelevant to its division, but it can be solved exactly. Going forward, we focus on the relationship Eq. (11a) between TSD and inoculum size for the simple birth process (red line), but emphasize that we would reach similar conclusions—at the price of analytic tractability—if we instead used the relationship for Poisson-distributed inocula and age-structured population growth (green triangles).

**Figure 3:**
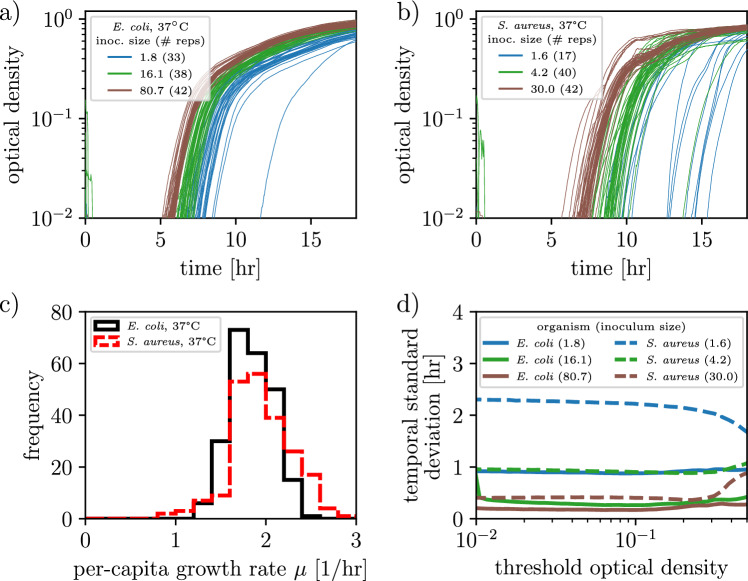
Empirical analyses of bacterial growth trajectories. (**a**, **b**) Measured abundance trajectories in *E. coli* and *S. aureus* as functions of time for different mean inoculum sizes. (**c**) Distribution of log-phase growth rates pooled across replicates and inoculum sizes, evaluated at an optical density of 0.03 (Methods). (**d**) Temporal standard deviations as functions of threshold optical density for different mean inoculum sizes.

### Bacterial growth experiments

To empirically test the relationship between TSD and inoculum size, we measured the growth of *E. coli* and *S. aureus*. At least 30 biological replicates were prepared for each inoculum size and grown over one or two days. Inoculum sizes were set by pipetting a dilute solution of bacteria growing in mid-log phase into a 96-well plate. Spot plating the same volume of this dilute solution established mean inoculum sizes and confirmed that inoculum sizes were Poisson distributed (Fig. S2). Bacterial abundance was inferred by measuring the optical density of each well every 2 min.

Figures 3a,b show representative subsets of abundance trajectories for *E. coli* and *S. aureus*, respectively. Bacteria grow exponentially until they reach an optical density of $\sim$0.2, then grow more slowly until the population enters stationary phase. During the exponential-growth phase, each individual’s growth rate is $\sim$2/hour ($\sim$20–30-min division times). Figure 3c shows the distribution of growth rates across replicates, calculated as the slope of the log-transformed optical-density time series evaluated at a threshold optical density of 0.03 (Methods). Measuring the growth rate $\mu$ at an optical density of 0.02 increases its value by 15%, while evaluating it at 0.05 decreases its value by 10%.

Lag phase, the time period during which bacteria do not divide after being transferred to a new environment, could in principle affect the temporal variation of a growing population^31–33^. However, we expect lag phase did not significantly impact our experiments: in our setup, bacteria in log phase (exponential growth) were back-diluted into fresh and otherwise-identical media so that their growth never halts (Methods). To check this expectation, for each inoculum size in Fig. 3a we calculated that the time required to reach an OD threshold of 0.03 ($\sim$ $10^7$ CFUs) assuming deterministic exponential growth with 1.8/hr growth rate and no lag phase exceeded the average empirically observed times by 30–60 minutes (Methods). A significant lag phase, by comparison, would imply that the first-passage time for the deterministic model without lag phase is shorter than the empirically observed time.

Equation (11a) predicts that the temporal standard deviation for the first-passage time to threshold $\Omega$ asymptotes to a constant value for $\Omega \gtrsim 50$. Figure 3d confirms this prediction: the TSD is approximately the same for threshold optical densities 0.01–0.3 (corresponding to millions to tens of millions of bacteria).

**Figure 4:**
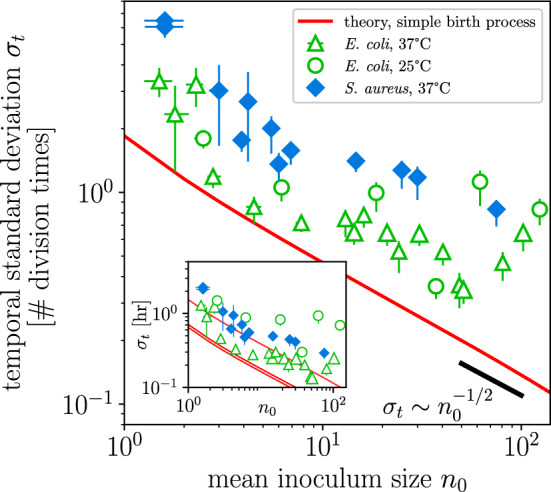
Temporal standard deviation scales inversely with the square root of the inoculum size in bacterial growth experiments. Temporal standard deviations for a total of 35 inoculum sizes in *E. coli* and *S. aureus*, in units of division times (at least 15 replicates per inoculum size, average 40). (inset) TSDs plotted in units of hours. The theoretical TSD for a given inoculum size [red line, Eq. (11a)] derived for the simple birth process lies under every experimental measurement (not a fit). Population-growth experiments were noisier than the limit of the simple birth process. Error bars indicate 68% confidence intervals of the mean (Methods). (inset) Red lines from top to bottom calculated with growth rates from *E. coli* at 25 °C, *E. coli* at 37 °C, and *S. aureus* at 37 °C; the shaded red regions take into account variation in measured growth rates (as shown in Fig. 3c), depicting theoretical TSDs for which growth rates differ by up to one standard error of the mean from their mean.

Bacterial growth experiments were performed for 35 inoculum sizes, yielding 1381 total growth curves. Figure 4 shows how TSDs depend on inoculum size in units of hours (inset) and in units of division times (main figure). An organism’s division time is defined as $\ln (2)/\mu$ for growth rate $\mu$: at 37 °C, *E. coli* and *S. aureus* have division times of $\sim$22 minutes, and at 25 °C *E. coli* has a division time of $\sim$50 minutes (Methods). Presenting the data in terms of division times rather than hours collapses the TSDs of *E. coli* at 25 °C onto the TSDs of *E. coli* at 37 °C in Fig. 4.

In the stochastic growth models considered in Fig. 2, noise in abundance trajectories is generated either by variability in the timing of division events or by variability in inoculum size. In practice, abundance trajectories are additionally buffeted by noise sources that the stochastic growth models do not account for. Most noise sources will make replicate trajectories more variable, thereby increasing their TSD: differing media conditions, temperature fluctuations, or lag phase could each act as a *dispersive* noise source. Other *focusing* noise sources, for example when mother and daughter cells have strongly anticorrelated division times, can reduce the variability among replicate trajectories^34^.

In Fig. 4, the temporal standard deviation predicted by the simple birth process (red line) lies below all 35 experimentally tested inoculum sizes (colored symbols). This, our main empirical result, provides strong experimental support for the relationship (11a) as a lower bound to the temporal variation of an exponentially growing population. The deviations between measured and predicted TSDs reflect noise sources that are not captured by the stochastic growth model, and indicate that dispersive noise sources outweigh any focusing ones.

### Accumulation of temporal variation

For the simple birth process, contributions to the temporal variance [Eq. (10)] fall off as the inverse square of the population size. This inverse-square trend is also numerically observed in exactly inoculated age-structured population-growth models (Fig. S3). For populations with Poisson-distributed inocula the stochastic process of inoculation spontaneously generates temporal variation. Thus, the largest contributions to temporal variation occur at small population sizes, which means that the growth rate at small population sizes should be made manifest in the noise.

Changing perspective from small population sizes to early times, we next quantify the time scale over which temporal variance accumulates in a growing population. We consider a two-step growth process. First, a population with inoculum size $n_0$ grows until a time *t* according to the simple birth process, yielding a distribution $P_t(n \, | \, n_0)$ over abundances *N*(*t*). Second, at time *t* population growth becomes deterministic and exponential (and hence this stage of growth does not contribute to the temporal variance). We define the random variable *T*[*N*(*t*)] to be the first-passage time for such deterministic exponential growth to reach a threshold $\Omega$ given that the inoculum size is a random variable *N*(*t*),

19 $$\begin{aligned} T[N(t)] = \frac{1}{\mu } \ln \left[ \Omega /N(t) \right] , \end{aligned}$$

where we assume the threshold $\Omega$ is much larger than any abundance *N*(*t*) before deterministic growth begins.

The mean $\langle N(t) \rangle$ and variance $\langle N(t)^2 \rangle - \langle N(t) \rangle ^2$ of the simple birth process are known [Eqs. (4) and (5)], so the variance of this first-passage-time distribution may be computed with the delta method (17a), yielding

20 $$\begin{aligned} \langle T[N(t)]^2 \rangle - \langle T[N(t)] \rangle ^2 = \frac{1}{\mu ^2 n_0}\left( 1 - e^{-\mu t} \right) + O\left( \frac{1}{n_0^2}\right) . \end{aligned}$$

For $t \gg 1/\mu$, this recovers to leading order the relationship (11b) for the simple birth process between temporal standard deviation and inoculum size. Strikingly, comparing Eq. (20) to Eq. (10) (which was derived for growth that exclusively obeys the simple birth process), after a single division time $\ln (2)/\mu$ the temporal variance reaches half of its asympotic value. Temporal variation is rapidly accumulated at early times (while populations are still small).

### Growth-rate inference

Rearranging Eq. (11a), for a given inoculum size $n_0$ and experimentally measured TSD $\sigma _t$ at large threshold $\Omega$, a noise-based estimate $\hat{\mu }$ of the growth rate is

21 $$\begin{aligned} \hat{\mu }&= \frac{1}{\sigma _t}\left[ \frac{1}{n_0^2} + \frac{1}{(n_0+1)^2} + \cdots + \frac{1}{(\Omega -1)^2}\right] ^{1/2}. \end{aligned}$$

This estimate assumes that growth follows the simple birth process and that no other sources of noise are present; any noise sources that are not accounted for by the stochastic growth model will bias this estimate. Figure 4 shows that measured TSDs were larger than the TSDs predicted by the simple birth process, which we interpret as the presence of dispersive noise sources that increased the variability of replicate trajectories. Due to this, in our case the estimated growth rate $\hat{\mu }$ will be a lower bound for the actual growth rate $\mu$.

Since most noise accumulates at small population sizes, the noise-based estimate $\hat{\mu }$ should be dominated by the growth rate at small population sizes. This meets an important need in microbial ecology experiments, which is to measure the growth rate of strains before they significantly change the media. Contemporary approaches quantify growth rates in small bacterial populations by directly observing the spatiotemporal dynamics of bacteria at sub-100nm spatial resolution, requiring cutting-edge microscopy and analysis methods^35,36^. By contrast, $\hat{\mu }$ depends exclusively on quantities that are straightforward to measure with standard microbiology lab equipment (namely, microplate readers and materials for colony-forming-unit counting assays). This noise-based technique opens the door to experiments that assess the characteristics of small cellular populations using standard optical-density measurements.

**Figure 5:**
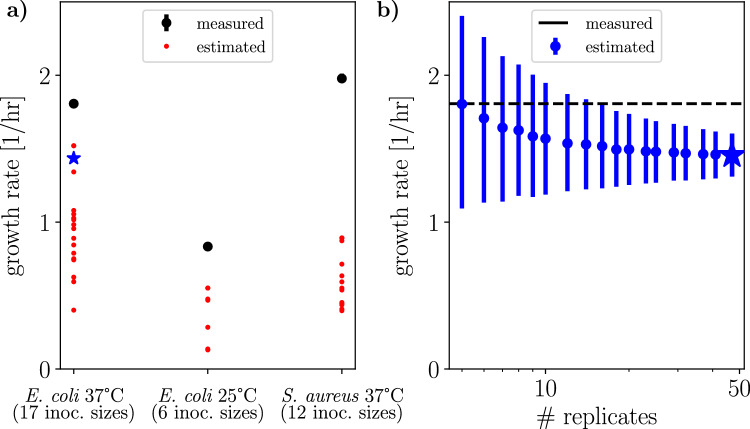
Noise-based estimation of growth rates. (**a**) Growth-rate estimates $\hat{\mu }$ for each organism, growth condition, and inoculum size, and assuming that growth follows a simple birth process, are plotted as red dots. Measured growth rates (black points, as in Fig. 3c) are calculated as the slopes of log-transformed abundance trajectories. (**b**) Precision of growth-rate inference, calculated by bootstrapping the abundance trajectories for *E. coli* at 37 °C with mean inoculum size 2.8 [blue star in (**a**)]. Error bars for measured (**a**) and estimated (**b**) growth rates show 68% confidence intervals from *n*=5000 bootstrap resamples per data point.

As a proof of concept, we applied this method to our noisy bacterial growth trajectories. Figure 5a compares the growth-rate estimate $\hat{\mu }$ for each organism, growth condition, and inoculum size to the measured growth rate (*i.e.*, slope of log-transformed optical-density time series) of each organism and growth condition. The measured rate exceeded the greatest of the estimates by 19% in *E. coli* at 37 °C, 51% in *E. coli* at 25 °C, and 71% in *S. aureus* at 37 °C. To probe how confidence in the estimation of $\hat{\mu }$ depends on the number of replicate growth trajectories, we bootstrap resampled a set of 47 abundance trajectories with mean inoculum size 2.8 in Fig. 5b.

### Desynchronization of division times

Finally we sought to understand when age-structured population growth becomes indistinguishable from the simple birth process, a crossover that helps to explain why TSDs of the two models have the same scaling behavior for large inoculum sizes in Fig. 4. This crossover occurs when growth-rate oscillations in the age-structured model (corresponding to initially synchronized division events) desynchronize, at which point the population grows at a constant exponential rate^37^.

In Supplementary Information Section C, we consider a deterministic age-structured population-growth model and apply Laplace-transform methods to determine the decay rate of growth-rate oscillations. For a division-time distribution with 25-min mean and 22% coefficient of variation, the growth dynamics of a single inoculum asymptote to pure exponential growth after $\sim$3 division cycles (Fig. S1). Our bacterial optical-density measurements have a resolution of 0.001 ($\sim$ $3 \times 10^5$ CFUs, corresponding to $\sim$18 division cycles), which suggests that such measurements cannot resolve any abundance oscillations predicted by age-structured growth models. Said another way, after a few division cycles one may approximate the growth dynamics of age-structured growth by a simple birth process.

We note that the deterministic age-structured model we consider ignores correlations between mother and daughter generation times, which have been empirically observed in bacteria^38^. Models that include cell-size control can extend the predicted persistence time of growth-rate oscillations^39^. In the future, time-lapse microscopy of entire bacterial populations could be used to directly observe the desynchronization of populations with small inoculum sizes.

## Discussion

Stochastic population growth, by its nature, produces a distribution of abundance trajectories over time^40^. For exponentially growing populations, the mean trajectory of this distribution contains information about the population growth rate, given by the slope of the log-transformed trajectory. We demonstrated in this paper that the temporal standard deviation is a second statistic that reports on the population growth rate. Temporal variation is especially informative when the birth rate is much larger than the death rate; temporal variation is less meaningful when populations fluctuate about a steady-state abundance or go extinct^26^.

Traditionally it has been difficult to measure the growth rate of bacteria at small population sizes without expensive microscopy equipment, since conventional optical-density measurements are unable to resolve growth at small scales^35,36,41,42^. Addressing this need, our noise-based inference method suggests that the temporal standard deviation at a large population threshold (easily calculated with optical-density measurements) can be related to the growth rate at small population sizes.

Temporal variation is a natural and useful description of noisy population growth. It does not depend on the conversion factor between optical density and CFUs, saving experimental effort. The population dynamics of colonizing species during microbiome assembly are stochastic^43^ and could be characterized in terms of temporal variation. The lower bound for the noise in *S. aureus* growth suggests a lower bound on the variation in times at which patients develop symptoms from the virulent hospital pathogen methycillin-resistant *S. aureus* (MRSA) following exposure^44^.

In an era of high-throughput biological experiments, noise-based analyses are becoming increasingly valuable. In this paper we found a signal in the noise that relates growth rate, inoculum size, and temporal standard deviation in exponentially growing systems. Leveraging this relationship, in well-controlled bacterial growth experiments we demonstrated a proof of concept for the noise-based inference of population growth rate, setting the stage for future statistical analyses of noisy population growth.

## Methods

### Bacterial growth experiments

Either *E. coli* strain MG1655 or *S. aureus* strain NCTC 8532 was grown overnight in lysogeny broth (LB), then back diluted 1:1000 and grown to a 600nm optical density (OD600) of 0.5. At this optical density bacterial growth is in mid-log phase. Serial dilutions were performed to obtain a culture with cell concentrations between 1 and 150 CFU per 2$\mu$L. This cell culture was subsequently used to inoculate bacterial growth experiments (*e.g.,* those in Fig. 3a,b) by pipetting 2$\mu$L of cell culture into 198 $\mu$L of LB media. Pipetting was performed with the Rainin Pipet-Lite Multi Pipette L8-20XLS+, accurate to $\pm 0.2 \mu$L. For each cell-culture concentration, 42 replicates were inoculated on the same 96-well plate to reduce variation, with 6 wells left as blank controls; each 96-well plate was inoculated with two sets of bacterial growth experiments. Plates were sealed with a “breathe-easy” with small holes poked in it to increase oxygen. Preparation and inoculation of 96-well plates was performed at 24.6 °C (room temperature). Preparing each batch of experiments (consisting of three 96-well plates) took $\sim$15 minutes from start to finish, with inoculations for each inoculum size spanning $\sim$3 min from start to finish.

Plates were grown in a Biotek Epoch 2 plate reader for 24 h at 37 °C (or 25 °C) with continuous orbital shaking. Optical-density readings at OD600 were taken every two or 3 min. When *E. coli* was grown at 25 °C, the time in the plate reader was extended to 48 h. By the Beer-Lambert law, bacterial population size and OD600 are linearly correlated in the sensitivity range of the plate reader (>0.01 OD)^45^. Optical-density measurements therefore serve as a proxy for bacterial population size.

### Measurement of inoculum size

For each concentration of cell culture, the distribution of the number of bacteria pipetted into each well of the 96-well plate (*i.e.,* the inoculum size) was inferred by spot plating identical volumes of cell culture on LB-agar plates^46^. Colonies were counted after 16 h of growth. For each concentration of cell culture, the inoculum size is roughly Poisson-distributed (Fig. S2). The mean $n_0$ of nonzero inoculum sizes is utilized in Figs. 3 and 4.

### Lag phase

For the three inoculum sizes in Fig. 3a we do not find evidence of a significant lag phase: the calculated time for a model of deterministic exponential growth with no lag phase to reach an optical density of 0.03 ($\sim$ $1.4\times 10^7$ CFUs) exceeded the mean observed time by 30 min for $n_0 = 80.7$; by 36 min for $n_0 = 16.1$; and by 59 min for $n_0 = 1.8$.

This analysis required a standard curve to convert optical density measurements to CFUs, measured by spot plating following serial dilution^47^. For this standard curve, measured optical densities spanned from 0.01 to 0.6, and measured CFUs spanned from $6 \times 10^6$ to $2 \times 10^8$. For each cell-culture concentration, measurements were performed for 7 biological replicates. Based on linear regression, an OD of 0.03 corresponds to $\sim$ $1.4\times 10^7$ CFUs.

### Bacterial strains

The MG1655 strain of *E. coli* (ATCC 700926) was obtained from the Broderick lab at Johns Hopkins University. The NCTC 8532 strain of *S. aureus* (ATCC 12600) was obtained from the Saleh lab at Johns Hopkins University. Cultures were obtained by streaking from glycerol stocks onto LB-agar plates and grown for 16 h at 37 °C.

### Criteria for omission of growth curves

Bacterial growth curves were omitted from analysis if: (i) a well was missing an air puncture, causing anerobic growth (3/1439 replicates omitted), (ii) a well was contaminated (2/1439 replicates omitted), or (iii) raw OD600 after 1 h of growth was above 0.125, indicating initial condensation or measurement error (47/1439 replicates omitted). In total, these exclusion criteria led to the omission of 4% (52/1439) of growth trajectories. Figure S4 shows all raw growth curves, with omitted curves in red.

### Removing optical-density background

The measurement background—corresponding to the light occluded by solution (not bacteria) in a well—was subtracted from each optical-density time-series. The background was calculated as the mean optical density at time 0 for each 96-well plate, and ranged from an optical density of 0.099–0.121. Figures 3a,b show representative background-subtracted optical-density measurements. For reference, empty dry wells yield optical-density measurements of 0.005.

### Growth-rate calculation

For a particular bacterial growth curve, the growth rate $\mu$ is determined by linearly regressing the log-transformed background-subtracted optical-density trajectory. Operationally, the growth rate at a given time $t_0$ is calculated as the slope of the best-fit line for the 30-min window centered at $t_0$. A single growth rate was calculated for each organism and growth condition, defined as the average growth rate across replicates and inoculum sizes evaluated at times $t_0$ when optical-density trajectories reach threshold optical density 0.03: *E. coli* at 37 °C grows at $\mu =1.8$/hr, *E. coli* at 25 °C grows at $\mu =0.8$/hr, and *S. aureus* at 37 °C grows at $\mu =2.0$/hr. The growth rate is relevant for plotting TSDs in units of division time in Fig. 4, since an organism’s division time is defined as $\ln (2)/\mu$.

### Population-growth models

For each population-growth model plotted in Fig. 2, a set of integer inoculum sizes ranging from 1 to 30 were simulated. Models with Poisson-distributed inocula used this integer inoculum size as the Poisson shape parameter; the subsequent zero-truncated Poisson distribution has a larger mean inoculum size, giving rise to non-integer mean inoculum sizes. The simple birth process with exact inoculation (red) and deterministic exponential growth with Poisson-distributed inocula (blue) were computed exactly with Eqs. (10a) and (S26), respectively.

The age-structured population-growth model with exact inoculation (gold) was simulated in an agent-based manner. Inoculated individuals were assumed to be at a random point along their division cycle, so their first division event was set to a random time uniformly drawn from [0, $(\ln 2)/\mu$]. Thereafter, after each division event, the two resulting individuals each randomly drew their next division time from a division-time distribution that is determined by a 20-stage growth process (in which reaching the next stage of development is a Poisson process with constant rate): specifically, this growth process yields a division-time distribution given by a chi-squared distribution $\chi ^2(40)$^4^, linearly rescaled so the mean division time was 25 min. Simulated TSDs were calculated at a threshold of 500 individuals.

Lastly, simple-birth-process simulations with Poisson-distributed inocula (purple) were performed by drawing 2000 inoculum sizes from an appropriate Poisson distribution, then performing stochastic simulations using the Python function birdepy.simulate.discrete. For each set of simulations (gold, green, purple), 95% confidence intervals were computed by bootstrapping using the Python function scipy.stats.bootstrap.

### Deterministic model of age-structured growth

Simulations of the deterministic age-structured population-growth model displayed in Fig. S1 were performed using the Mathematica functions TransferFunctionModel, TransferFunctionPoles, and NInverseLaplaceTransform.

### Software

Analyses were performed with Python (version 3.9.7) and Mathematica (version 12.1.0.0).

### Supplementary Information

Supplementary Information.

## Data Availability

Raw data from bacterial growth experiments and software that recreates the main text figures are available online at GitHub: https://github.com/erijones/intrinsic_variation.
