# Supplementary material for: First-passage-time statistics of growing microbial populations carry an imprint of initial conditions
Source: Sci Rep. 2023 Dec 4;13:21340. doi: 10.1038/s41598-023-48726-w (PMC10696051; doi:10.1038/s41598-023-48726-w)
Supplement: Supplementary file 1 — Supplementary Information. [file 41598_2023_48726_MOESM1_ESM.pdf]

Supplementary Information: First-passage-time statistics of growing microbial populations carry an imprint of initial conditions

Eric W. Jones[1], Joshua Derrick[2], Roger Nisbet[3], Will Ludington[2,4], and David A. Sivak[1]
[1]*Department of Physics, Simon Fraser University*
[2]*Department of Biological Sciences and Engineering,*
Carnegie Institution for Science, Baltimore, MD 21218, USA
[3]*Department of Ecology, Evolution, and Marine Biology, University of California, Santa Barbara and*
[4]*Department of Biology, Johns Hopkins University, Baltimore, MD 21218*

Contents

Section A: First-passage-time distribution of the simple birth process 2

Section B: Mean and variance of the first-passage-time distribution for the simple birth process 4

Section C: Deterministic age-structured population growth 6

References 11

Section A: First-passage-time distribution of the simple birth process

For a simple birth process with with probability $P_t(n\,|\,n_0)$ of a population consisting of n individuals at time t given an inoculum size of n_0, the reaction probability $R_\Omega(t\,|\,n_0)$ that at time t the population size is greater than or equal to population threshold Ω is

$$R_\Omega(t\,|\,n_0) = 1 - \sum_{i=n_0}^{\Omega-1} P_t(i\,|\,n_0). \tag{S1}$$

Since abundance trajectories are monotonic, the reaction probability is also related to the first-passage-time probability $P_\Omega^{\mathrm{FP}}(t\,|\,n_0)$ of times t at which an abundance trajectory first reaches Ω individuals,

$$R_\Omega(t\,|\,n_0) = \int_0^t P_\Omega^{\mathrm{FP}}(\tau\,|\,n_0)\,\mathrm{d}\tau. \tag{S2}$$

By the fundamental theorem of calculus, the first-passage-time distribution $P_\Omega^{\mathrm{FP}}(t\,|\,n_0)$ is related to the solution $P_t(n\,|\,n_0)$ of the simple birth process:

$$P_n^{\mathrm{FP}}(t\,|\,n_0) = -\sum_{i=n_0}^{n-1} \frac{\mathrm{d}P_t(i\,|\,n_0)}{\mathrm{d}t} \tag{S3a}$$

$$= \mu(n-n_0)\binom{n-1}{n_0-1} e^{-\mu n_0 t}(1-e^{-\mu t})^{n-n_0-1}. \tag{S3b}$$

Next, we prove that Eqs. (S3a) and (S3b) are equal. Recall that

$$P_t(n\,|\,n_0) = \frac{(n-1)!}{(n_0-1)!(n-n_0)!} e^{-\mu n_0 t}(1-e^{-\mu t})^{n-n_0}, \tag{S4}$$

so by Eq. (S3a),

$$P_n^{\mathrm{FP}}(t\,|\,n_0) = \frac{\mathrm{d}}{\mathrm{d}t}\left[-e^{-\mu n_0 t}(1-e^{-\mu t})^{-n_0} \sum_{i=n_0}^{n-1} \binom{i-1}{n_0-1}(1-e^{-\mu t})^i \right] \tag{S5a}$$

$$= \mu e^{-\mu n_0 t}(1-e^{-\mu t})^{-n_0-1} \times \left[\sum_{i=n_0}^{n-1} \binom{i-1}{n_0-1}(n_0 - ie^{-\mu t})(1-e^{-\mu t})^i \right] \tag{S5b}$$

$$= \mu e^{-\mu n_0 t}(1-e^{-\mu t})^{-n_0-1} \times \frac{1}{(n_0-1)!}\left[\sum_{i=n_0}^{n-1} \frac{(i-1)!}{(i-n_0)!}(n_0 - ie^{-\mu t})(1-e^{-\mu t})^i \right]. \tag{S5c}$$

To evaluate the quantity in square brackets, we proceed by induction. Define

$$a_i \equiv \binom{i-1}{n_0-1}(n_0 - ie^{-\mu t})(1-e^{-\mu t})^i. \tag{S6}$$

We will show that

$$S_{n_0,n} \equiv \sum_{i=n_0}^{n-1} a_i = \binom{n-1}{n_0-1}(1-e^{-\mu t})^n (n-n_0). \tag{S7}$$

First, the base case is satisfied:

$$S_{n_0,n_0+1} = a_{n_0} = n_0(1-e^{-\mu t})^{n_0+1}. \tag{S8}$$

Next we assume

$$S_{n_0,n-1} = \binom{n-2}{n_0-1}(1-e^{-\mu t})^{n-1}(n-1-n_0) \tag{S9}$$

and prove the inductive step

$$S_{n_0,n} = \binom{n-1}{n_0-1}(1-e^{-\mu t})^{n}(n-n_0). \tag{S10}$$

We find

$$\begin{aligned}
S_{n_0,n} &= S_{n_0,n-1} + a_{n-1} && \text{(S11a)}\\
&= \binom{n-2}{n_0-1}(1-e^{-\mu t})^{n-1}(n-1-n_0) + \binom{n-2}{n_0-1}(n_0-(n-1)e^{-\mu t})(1-e^{-\mu t})^{n_0-1} && \text{(S11b)}\\
&= \binom{n-2}{n_0-1}(1-e^{-\mu t})^{n-1}\left[(n-1-n_0)+(n_0-(n-1)e^{-\mu t})\right] && \text{(S11c)}\\
&= \binom{n-2}{n_0-1}(1-e^{-\mu t})^{n-1}\left[n-1-ne^{-\mu t}+e^{-\mu t}\right] && \text{(S11d)}\\
&= \binom{n-2}{n_0-1}(1-e^{-\mu t})^{n}(n-1) && \text{(S11e)}\\
&= \binom{n-1}{n_0-1}(1-e^{-\mu t})^{n}(n-n_0), && \text{(S11f)}
\end{aligned}$$

as required. Therefore,

$$\begin{aligned}
P_n^{\mathrm{FP}}(t\,|\,n_0) &= \mu e^{-\mu n_0 t}(1-e^{-\mu t})^{-n_0-1}S_{n_0,n} && \text{(S12a)}\\
&= \mu(n-n_0)\binom{n-1}{n_0-1}e^{-\mu n_0 t}(1-e^{-\mu t})^{n-n_0-1}, && \text{(S12b)}
\end{aligned}$$

in agreement with Eq. (S3b).

Section B: Mean and variance of the first-passage-time distribution for the simple birth process

The mean first-passage time is

$$
\begin{aligned}
\langle t\rangle_{n\,|\,n_0} &= \int_0^\infty t\, P_n^{\mathrm{FP}}(t\,|\,n_0)\,\mathrm{d}t && \text{(S13a)}\\
&= \mu(n-n_0)\binom{n-1}{n_0-1}\int_0^\infty t e^{-\mu n_0 t}(1-e^{-\mu t})^{n-n_0-1}\,\mathrm{d}t && \text{(S13b)}\\
&= \mu(n-n_0)\binom{n-1}{n_0-1}\sum_{k=0}^{n-n_0-1}\binom{n-n_0-1}{k}(-1)^k\int_0^\infty t e^{-\mu(n_0+k)t}\,\mathrm{d}t && \text{(S13c)}\\
&= \mu(n-n_0)\binom{n-1}{n_0-1}\sum_{k=0}^{n-n_0-1}\binom{n-n_0-1}{k}(-1)^k\left[\frac{-e^{-\mu(n_0+k)t}(1+\mu(n_0+k)t)}{\mu^2(n_0+k)^2}\right]_0^\infty && \text{(S13d)}\\
&= \mu(n-n_0)\binom{n-1}{n_0-1}\sum_{k=0}^{n-n_0-1}\binom{n-n_0-1}{k}\frac{(-1)^k}{\mu^2(n_0+k)^2} && \text{(S13e)}\\
&= \frac{1}{\mu}\left(\frac{1}{n_0}+\frac{1}{n_0+1}+\cdots+\frac{1}{n-1}\right), && \text{(S13f)}
\end{aligned}
$$

where the last equality follows from the identity Eq. (S17b). Similarly,

$$
\begin{aligned}
\langle t^2\rangle_{n\,|\,n_0} &= \int_0^\infty t^2\, P_n^{\mathrm{FP}}(t\,|\,n_0)\,\mathrm{d}t && \text{(S14a)}\\
&= \mu(n-n_0)\binom{n-1}{n_0-1}\sum_{k=0}^{n-n_0-1}\binom{n-n_0-1}{k}(-1)^k\int_0^\infty t^2 e^{-\mu(n_0+k)t}\,\mathrm{d}t && \text{(S14b)}\\
&= \mu(n-n_0)\binom{n-1}{n_0-1}\sum_{k=0}^{n-n_0-1}\binom{n-n_0-1}{k}(-1)^k\left[\frac{-e^{-\mu(n_0+k)t}(2+2\mu(n_0+k)t+\mu^2(n_0+k)^2t^2)}{\mu^3(n_0+k)^3}\right]_0^\infty && \text{(S14c)}\\
&= 2\mu(n-n_0)\binom{n-1}{n_0-1}\sum_{k=0}^{n-n_0-1}\binom{n-n_0-1}{k}\frac{(-1)^k}{\mu^3(n_0+k)^3} && \text{(S14d)}\\
&= \frac{1}{\mu^2}\left[\left(\frac{1}{n_0}+\frac{1}{n_0+1}+\cdots+\frac{1}{n-1}\right)^2+\left(\frac{1}{n_0^2}+\frac{1}{(n_0+1)^2}+\cdots+\frac{1}{(n-1)^2}\right)\right], && \text{(S14e)}
\end{aligned}
$$

where the last equality follows from the identity Eq. (S18c). Thus, the temporal variance $\sigma_t^2 \equiv \langle t^2\rangle - \langle t\rangle^2$ is

$$
\sigma_t^2 = \frac{1}{\mu^2}\left(\frac{1}{n_0^2}+\frac{1}{(n_0+1)^2}+\cdots+\frac{1}{(n-1)^2}\right). \tag{S15}
$$

To derive the identities Eqs.(S17b) and (S18c), start from the identify [1]

$$
\sum_{k=0}^{n}\binom{n}{k}\frac{(-1)^k}{k+x} = \left[x\binom{n+x}{n}\right]^{-1}. \tag{S16}
$$

Differentiating with respect to x yields

$$
\begin{aligned}
\sum_{k=0}^{n}\binom{n}{k}\frac{(-1)^k}{(k+x)^2} &= -\frac{\mathrm{d}}{\mathrm{d}x}\left[\frac{n!}{x(x+1)\cdots(x+n)}\right] && \text{(S17a)}\\
&= \frac{n!}{x(x+1)\cdots(x+n)}\left(\frac{1}{x}+\frac{1}{x+1}+\cdots+\frac{1}{x+n}\right). && \text{(S17b)}
\end{aligned}
$$

Differentiating the identity Eq. (S17b) again gives

$$2\sum_{k=0}^{n}\binom{n}{k}\frac{(-1)^k}{(k+x)^3} = \frac{\mathrm{d}^2}{\mathrm{d}x^2}\left[\frac{n!}{x(x+1)\cdots(x+n)}\right] \tag{S18a}$$

$$= \frac{n!}{x(x+1)\cdots(x+n)}\left(\frac{1}{x}+\frac{1}{x+1}+\cdots+\frac{1}{x+n}\right)^2 \tag{S18b}$$

$$+ \frac{n!}{x\cdots(x+n)}\left(\frac{1}{x^2}+\frac{1}{(x+1)^2}+\cdots+\frac{1}{(x+n)^2}\right). \tag{S18c}$$

These two identities provide the simplifications needed for Eqs. (S13f) and (S14e)

Section C: Deterministic age-structured population growth

Organismal division is intricately choreographed and can often be broken down into discrete stages [2]. Here we examine deterministic age-structured population growth models in which division-time distributions describe the timing of division events. In particular, with Laplace-transform methods we characterize the desynchronization of initially synchronized division events.

Let $n(a,t)\,\mathrm{d}a$ be the number of individuals aged between a and $a + \mathrm{d}a$ at time t (where age is defined as elapsed time since previous division), and assume individuals divide with propensity $\beta(a)$. Population dynamics are governed by the PDE [2, 3]

$$\frac{\partial n}{\partial t} + \frac{\partial n}{\partial a} + \beta(a) n = 0, \tag{S19}$$

together with the renewal condition that describes how individuals divide,

$$R(t) \equiv n(0,t) = 2\int_0^\infty \beta(a) n(a,t)\mathrm{d}a, \tag{S20}$$

and the initial condition

$$n(a,0) = n_0(a), \tag{S21}$$

where $R(t)$ is the recruitment rate of newly divided individuals at time t (*i.e.,* the rate at which age 0 individuals enter the population, or roughly twice the growth rate of the simple birth process).

A formal solution to Eq. (S19) is

$$n(a,t) = \begin{cases} R(t-a)S(a) \text{ for } t > a \\ n_0(a-t)\tilde{S}(a,t) \text{ for } t \le a \end{cases}, \tag{S22}$$

where the "survival" function $S(a) \equiv \exp\left[-\int_0^a \beta(u)\,\mathrm{d}u\right]$ is the proportion of individuals that survive to age a before dividing, and the modified survival function $\tilde{S}(a,t) \equiv \exp\left[-\int_{a-t}^a \beta(u)\,\mathrm{d}u\right]$ is the proportion of individuals that survive to age a before dividing given that they existed and were undivided at age $a - t$. It is also convenient to define a normalized division-time distribution $\mathrm{P}_{\mathrm{DT}}(a) \equiv \beta(a)S(a)$. Provided no deaths occur, these functions are related according to

$$S(a) = 1 - \int_0^a \mathrm{P}_{\mathrm{DT}}(u)\,\mathrm{d}u. \tag{S23}$$

From Eqs. (S20) and (S22),

$$\begin{aligned} R(t) &= \underbrace{2\int_0^t R(t-a)\beta(a)S(a)\,\mathrm{d}a}_{\text{created after } t=0} + \underbrace{2\int_t^\infty n_0(a-t)\beta(a)\tilde{S}(a,t)\,\mathrm{d}a}_{\text{from inoculum}} \\ &= 2\int_0^t R(t-a)\mathrm{P}_{\mathrm{DT}}(a)\,\mathrm{d}a + F(t), \end{aligned} \tag{S24}$$

with $F(t) \equiv 2\int_t^\infty n_0(a-t)\beta(a)\tilde{S}(a,t)\,\mathrm{d}a$ giving contributions to the recruitment rate from individuals that have not divided since inoculation.

In the special case where the inoculum consists of N_0 newly divided cells, $n_0(a) = N_0\delta(a)$ for Dirac delta function $\delta(a)$, and $F(t) = 2N_0\mathrm{P}_{\mathrm{DT}}(t)$. From Eqs. (S20) and (S24), the total population size is

$$N(t) = \int_0^\infty n(a,t)\,\mathrm{d}a = \underbrace{\int_0^t R(t-a)S(a)\,\mathrm{d}a}_{\text{created after } t=0} + \underbrace{\int_t^\infty n_0(a-t)\tilde{S}(a,t)\,\mathrm{d}a}_{\text{from inoculum}}, \tag{S25}$$

which in this special case simplifies to

$$N(t) = \int_0^t R(t-a)S(a)\,\mathrm{d}a + N_0 S(t). \tag{S26}$$

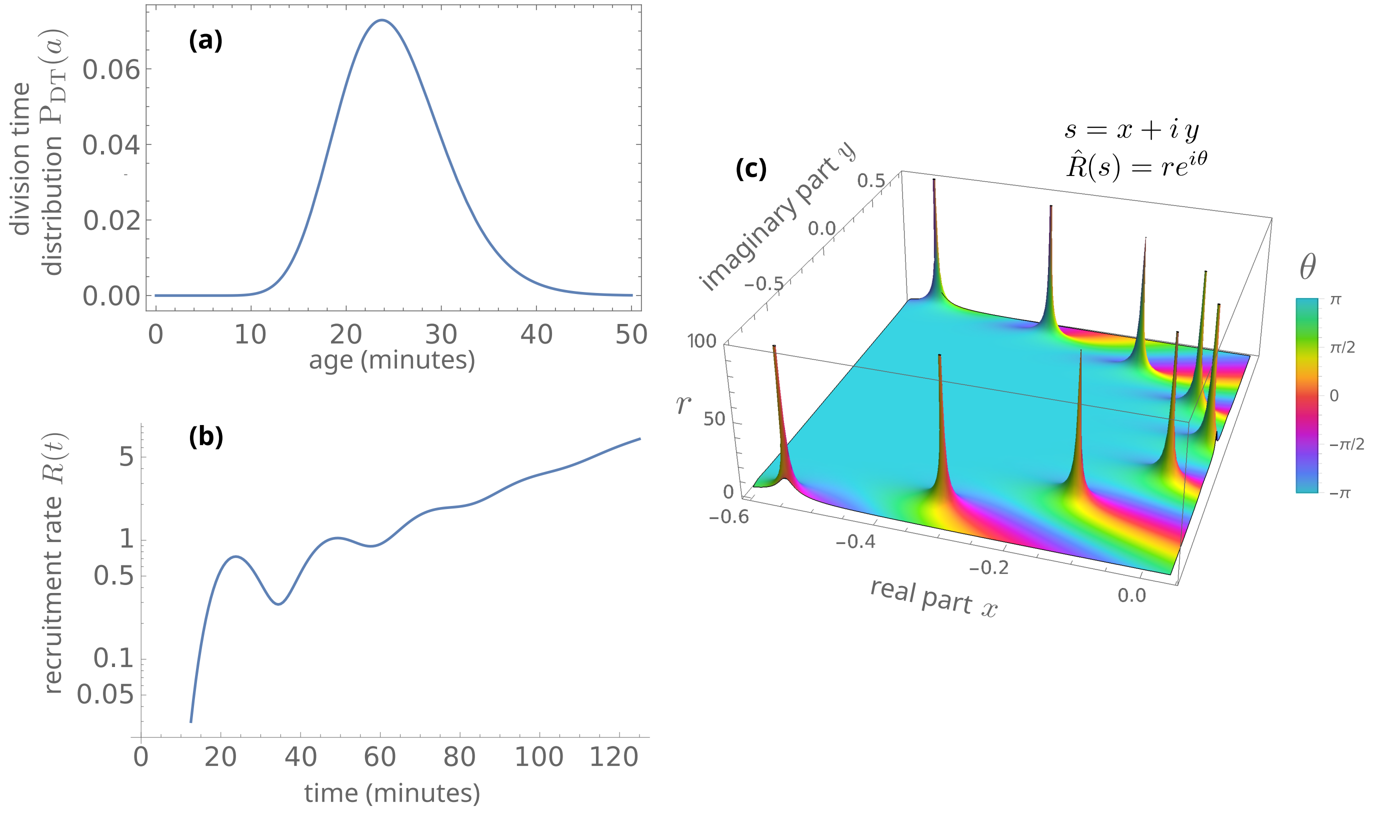

FIG. S1: **Characterization of age-structured population growth.** (a) Division-time distribution for a 20-stage population-growth model given by a chi-squared distribution $\chi^2(2k)$ with $k = 20$ (22% coefficient of variation), linearly rescaled such that the mean division time is 25 minutes. (b) Recruitment rate $R(t)$ contains transient oscillations that decay after a few division times. (c) Poles of the Laplace transformed recruitment rate $\hat{R}(s)$ in an age-structured population-growth model, plotted in the complex plane. The pole with the largest real part has a positive real part and determines the long-run population-growth rate; the complex-conjugate pairs contribute to transient oscillations. The location of the three poles nearest the right-hand edge are used to compute the coherence number Eq. (S31).

The dynamics of this age-structured population therefore depend entirely on the division propensity $\beta(a)$, by way of the survival function $S(a)$ and the division-time distribution $\mathrm{P_{DT}}(a)$.

Laplace transforming Eq. (S24) yields

$$\hat{R}(s) = 2\hat{R}(s)\hat{\mathrm{P}}_{\mathrm{DT}}(s) + \hat{F}(s), \tag{S27}$$

implying that

$$\hat{R}(s) = T(s)\hat{F}(s), \tag{S28}$$

with $T(s) \equiv 1/[1 - 2\hat{\mathrm{P}}_{\mathrm{DT}}(s)]$. The transfer function $T(s)$ describes the mapping in the complex s-plane from both the initial distribution of ages in the population and the division-time distribution to the solution of the dynamical system. The explicit time series for a specified initial condition is obtained by inverse Laplace transformation, a task slightly simplified in the special case where the initial population consists of newly divided cells, for which $\hat{F}(s)= 2N_0\hat{\mathrm{P}}_{\mathrm{DT}}(s)$.

Following Kendall's 1948 seminal work [4], we consider a class of age-structured models in which the division-time distribution for a k-stage population-growth model is given by a chi-squared distribution with $2k$ degrees of freedom, $\mathrm{P_{DT}}(a) = \chi^2(2k)$. The solution has the form

$$R(t) = \sum_{\text{all poles } i} c_i \exp(s_i t), \tag{S29}$$

where the coefficients c_i depend on the initial age distribution, and the exponents s_i are the locations in the complex s-plane of the poles of the transfer function. The pole s_0 with the largest real part determines the long-run population-

growth rate:

$$R(t) = c_0 \exp(s_0 t) \left(1 + \sum_{i \neq 0} \frac{c_i}{c_0} \exp[(s_i - s_0)t] \right) , \tag{S30}$$

where the expression in parentheses approaches 1 as $t \to \infty$. The subdominant poles $s_{1,2}$ are typically a complex-conjugate pair and characterize the approach to asymptotic exponential growth. Defining $s_{1,2} \equiv \sigma \pm i\omega$ for real σ, the leading terms in the summation in Eq. (S30) are proportional to $\exp[(\sigma - s_0)t] \cos(\omega t - \phi)$, where ϕ sets the phase of any transient oscillations. The period of any transient oscillations is $2\pi/\omega$. For all cases we explored, this period is very close to the mean division time.

The transient decays by a factor of e over a time interval $1/(s_0 - \sigma)$. We define the *coherence number* n_c as the number of oscillations before the transient decays by a factor of e,

$$n_c \equiv \frac{\omega}{2\pi(s_0 - \sigma)}. \tag{S31}$$

The coherence number measures the rate at which intrinsic variability desynchronizes initially synchronized division events, and therefore informs the point at which detailed non-Markovian models may be approximated by coarse-grained Markovian models like the simple birth process. Accordingly, it takes $\log_e 10 \times n_c \approx 2.3 n_c$ oscillations in order for the transient to drop to 10% of its original magnitude.

Figure S1 illustrates these concepts for the "deterministic skeleton" [5] of the stochastic age-structured model used in Fig. 2, which describes the deterministic and incremental development of individuals until division. It shows the division-time distribution for a 20-stage population-growth model with a mean division time of 25 minutes (Fig. S1a), the growth rate over time (Fig. S1b), and the leading poles (*i.e.,* those with largest real part) of the transfer function (and equivalently of $R(s)$) (Fig. S1c). The coherence number for this system is $n_c = 1.01$, implying that the approach to exponential growth (when the transients have dropped to 10% of their original magnitude) requires ~2.3 cell division cycles.

We conclude that the dynamics of the 20-stage model (with initially oscillatory growth rates) approach the dynamics of the simple birth process (with growth rates proportional to population size) after a few division cycles.

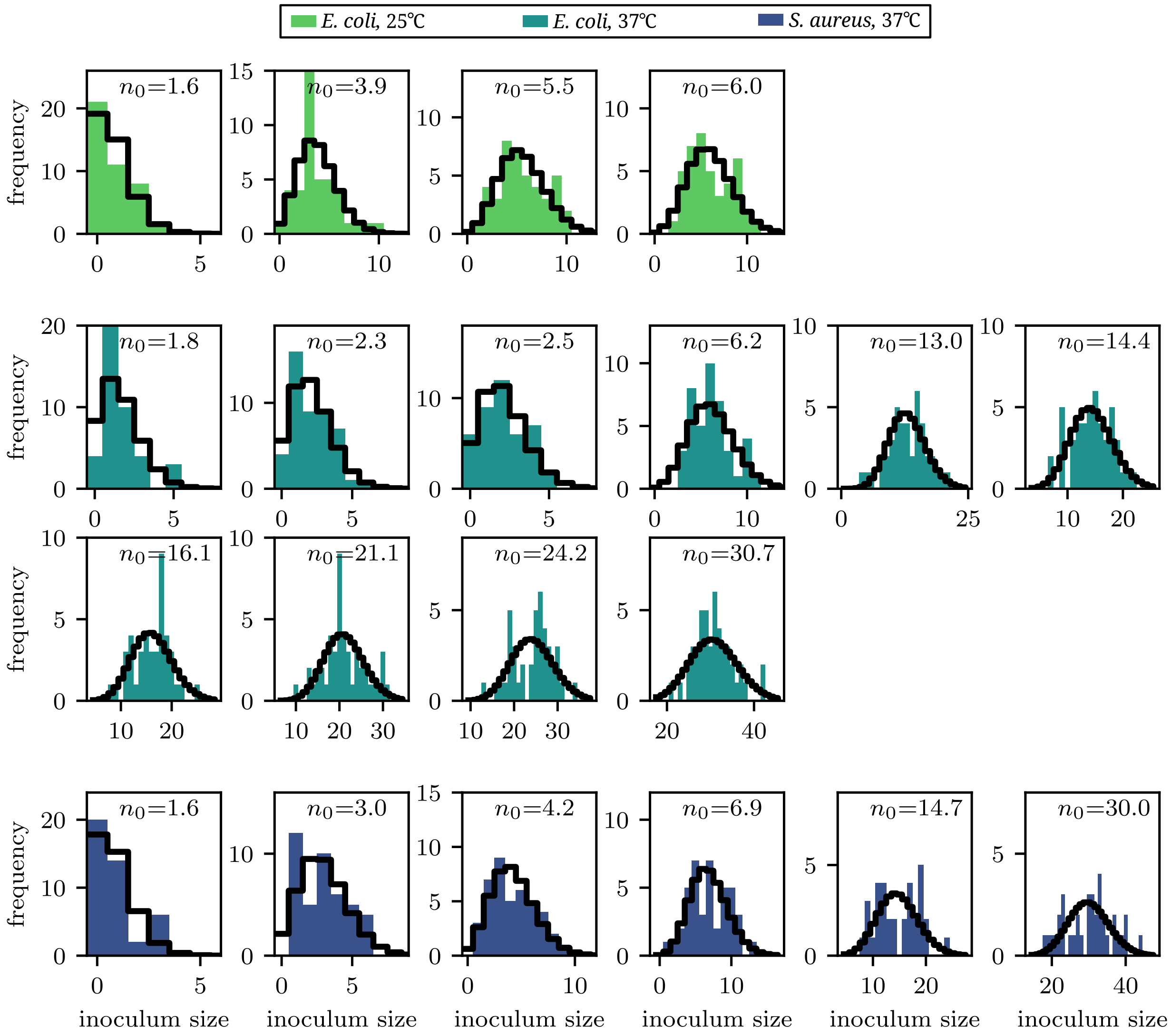

FIG. S2: **Inoculum sizes are roughly Poisson-distributed.** Distribution of inoculum sizes (filled histogram), measured by spot plating, for 20 cell cultures of varying concentrations (Methods). Black: theoretical Poisson distribution for the measured mean inoculum size. Spot-plating experiments were performed for each organism and growth condition, as indicated by the legend. For each distribution we report the zero-truncated mean abundance n_0, plotted in Figure 4.

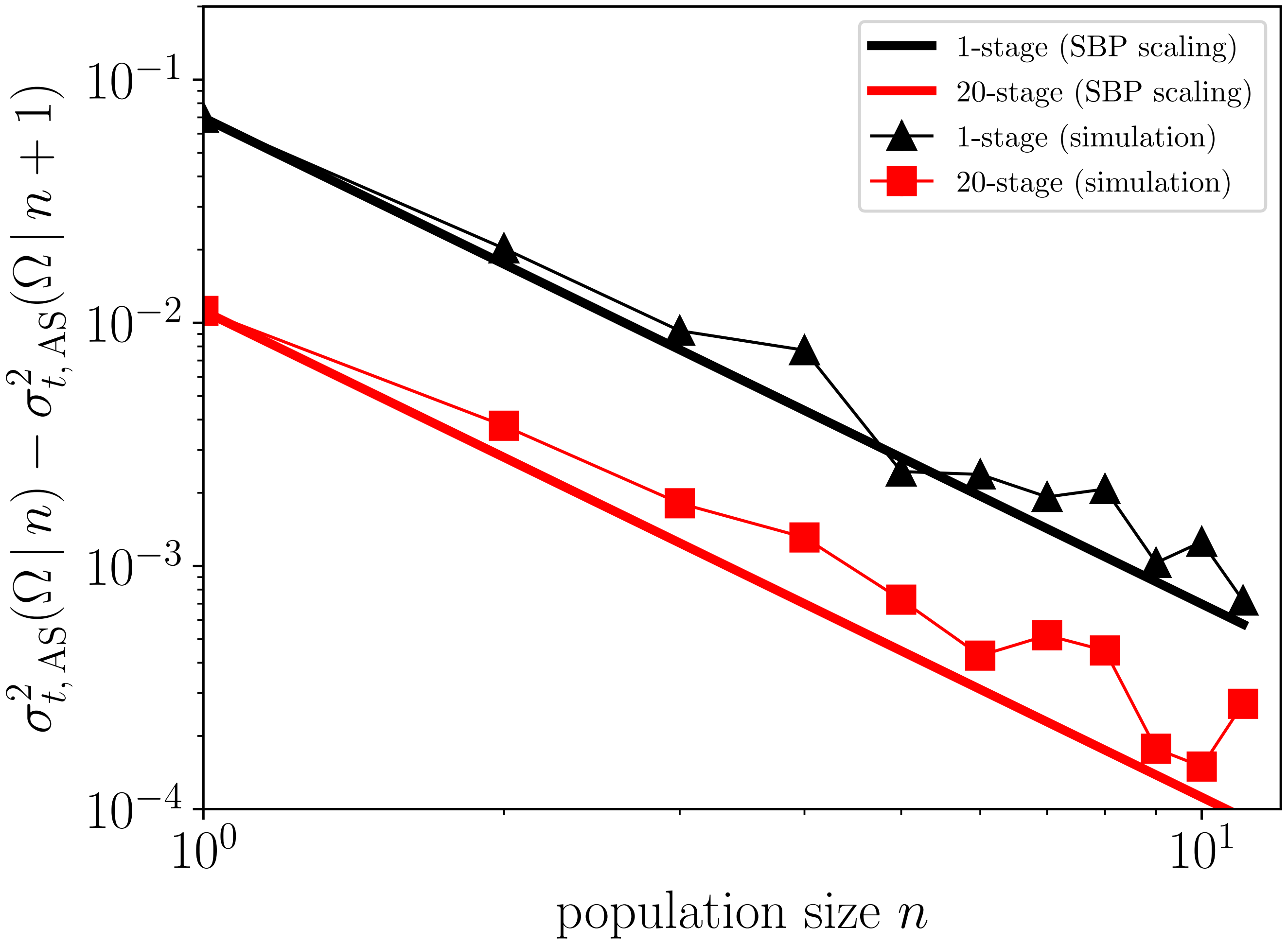

FIG. S3: **Contributions to the temporal variance across population sizes for age-structured population models.** Difference in asymptotic (large-Ω) temporal variance $\sigma^2_{t,\mathrm{AS}}(\Omega\,|\,n_0)$ between inoculum sizes n and $n+1$ (*i.e.*, the reduction in the asymptotic temporal variance by starting with one more individual) for age-structured population growth. Points are from 1-stage and 20-stage stochastic age-structured population models (Methods) with 20,000 trajectories. Temporal variances are evaluated at a threshold population size $\Omega = 500$. 95% confidence intervals are smaller than symbols. In the simple birth process, the temporal variance [Eq. (10)] is a sum with summands that scale as $1/n^2$. Solid lines depict this $1/n^2$ scaling, starting from the contribution to the asymptotic temporal variance of a single individual (graphically, a $1/n^2$ power law starting from the $n = 1$ data point). Thin lines are a guide to the eye.

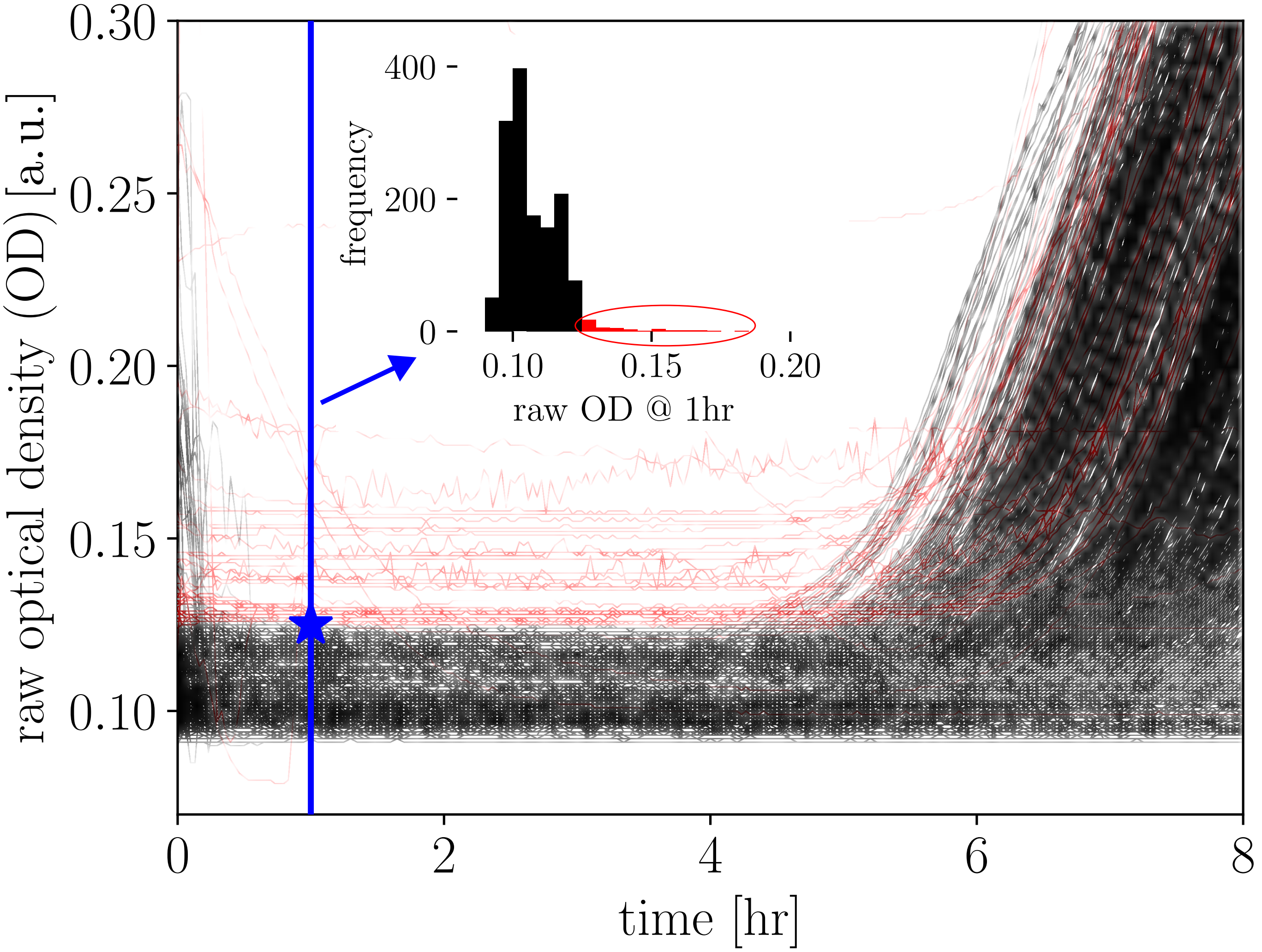

FIG. S4: **Raw optical-density measurements from all 1434 bacterial growth experiments, with 47 excluded growth curves shown in red.** Growth curves with a raw optical density (*i.e.*, before the background has been subtracted) greater than 0.125 at 1 hour post inoculation (indicated by the blue star and line) were omitted from subsequent analysis, and are indicated in red. Including every growth curve marginally increases TSDs (*e.g.*, in Fig. 4), as outliers inflate trajectory spread. (inset) Histogram of raw optical density at 1 hour post inoculation; the circled red bins indicate the ∼4% of trajectories that were excluded from subsequent analysis.

[1] D. E. Knuth, *The Art of Computer Programming, Volume 1* (Bulletin of the American Mathematical Society, 1997).
[2] B. Keyfitz and N. Keyfitz, Mathematical and Computer Modelling **26**, 1 (1997).
[3] R. Nisbet and W. Gurney, Mathematical Ecology: An Introduction pp. 95–115 (1986).
[4] D. G. Kendall, Biometrika **35**, 316 (1948).
[5] K. Higgins, A. Hastings, J. N. Sarvela, and L. W. Botsford, Science **276**, 1431 (1997).
